# Longitudinal changes in vitamin D concentrations and the association with type 2 diabetes mellitus: the Tromsø Study

**DOI:** 10.1007/s00592-022-02001-y

**Published:** 2022-12-02

**Authors:** Giovanni Allaoui, Charlotta Rylander, Ole-Martin Fuskevåg, Maria Averina, Tom Wilsgaard, Magritt Brustad, Rolf Jorde, Vivian Berg

**Affiliations:** 1 Department of Laboratory Medicine, Diagnostic Clinic, University Hospital of North – Norway, 9038 Tromsø, Norway; 2 Department of Medical Biology, Faculty of Health Sciences, UiT-The Arctic University of Norway, 9037 Tromsø, Norway; 3 Department of Community Medicine, Faculty of Health Sciences, UIT-The Arctic University of Norway, 9037 Tromsø, Norway; 4 Department of Clinical Medicine, Tromsø Endocrine Research Group, Uit-The Arctic University of Norway, 9037 Tromsø, Norway; 5 The Public Dental Health Service Competence Centre of Northern Norway (TkNN), 9019 Tromsø, Norway

**Keywords:** 25 Hydroxyvitamin D 3, 25 Hydroxyvitamin D 2, Longitudinal survey, Type 2 diabetes mellitus

## Abstract

**Aim:**

We aimed to investigate the relationship between pre- and post-diagnostic 25-hydroxyvitamin D (25(OH)D) concentrations and type 2 diabetes (T2DM) over a period of 30 years in individuals who developed T2DM compared to healthy controls.

**Methods:**

This case–control study included 254 participants with blood samples collected at five different time-points (T1–T5) between 1986 and 2016. Of the 254 participants, 116 were diagnosed with T2DM between T3 and T4, and were considered cases; the remaining 138 were controls. Linear mixed regression models were used to examine pre- and post-diagnostic changes in 25(OH)D concentrations, and logistic regression was used to examine associations between these concentrations and T2DM at each time-point.

**Results:**

25(OH)D concentrations at different time-points and the longitudinal change in concentrations differed between cases and controls, and by sex. For women, each 5-nmol/l increase in 25(OH)D concentrations was inversely associated with T2DM at T3 (odds-ratio, OR, 0.79), whereas for men, this same increase was positively associated with T2DM at T1 (OR 1.12). Cases experienced a significant decrease in pre-diagnostic 25(OH)D concentrations (*p* value < 0.01 for women, *p* value = 0.02 for men) and a significant increase in post-diagnostic 25(OH)D concentrations (*p* value < 0.01 for women, *p* value = 0.01 for men). As such, each 1-unit increase in month-specific *z*-score change between T1 and T3 was significantly inversely associated with T2DM (OR 0.51 for women, OR 0.52 for men), and each such increase between T3 and T5 was significantly positively associated with T2DM in women (OR 2.48).

**Conclusions:**

25(OH)D concentrations seem to be affected by disease progression and type 2 diabetes diagnosis.

**Supplementary Information:**

The online version contains supplementary material available at 10.1007/s00592-022-02001-y.

## Introduction

The prevalence of type 2 diabetes (T2DM) has increased over the past decades, and this increase is projected to continue [1, 2]. As part of an effort to improve the prevention and treatment of T2DM, there has been an increased interest in assessing risk factors as potential targets for interventions; one such risk factor is vitamin D [3, 4]. Vitamin D is metabolised in the liver to 25-hydroxyvitamin D (25(OH)D) and then further metabolised in the kidneys to the biologically active form, 1,25-dihydroxyvitamin D (1,25(OH)2D) [5, 6]. Vitamin D status is mainly based on 25(OH)D concentration, due to its longer half-life; 1,25(OH)2D is not generally used, as it is tightly regulated by the kidneys and levels are often normal in vitamin D-deficient individuals [5, 7]. The main function of vitamin D is to regulate calcium and phosphate levels in bone metabolism, but may also be involved in glycemic control, beta cell protection, and insulin secretion and resistance as vitamin D receptors are present in pancreatic beta cells and in target tissues for insulin, such as the liver, skeletal muscle, and adipose tissue [8–11].

Longitudinal studies have reported significant associations between vitamin D deficiency and increased risk of T2DM [12, 13]. Repeated measurements of vitamin D in the same individuals who received healthy lifestyle advice demonstrated that improved vitamin D status over time was associated with reduced risk of T2DM over a mean follow-up of 1.1–2.7 years [14, 15]. In contrast, vitamin D supplements have not proven to improve glycaemic control or reduce the risk of T2DM; hence, causality has not been established [16]. Pittas et al. suggests that the difficulties in assessing causality between vitamin D and T2DM might be due to the slow progression, complexity, and heterogeneity of the disease [16]. Accordingly, vitamin D levels are associated with several other risk factors for T2DM, such as age, body weight, and physical activity (as a proxy for sun exposure and energy expenditure); hence, associations between vitamin D and T2DM may be confounded by these risk factors [8, 17]. Repeated measurements yield more accurate measures of exposures and confounders than a single baseline measurement [18], and the Tromsø Study provides a unique opportunity to explore the longitudinal relationship between vitamin D, risk factors, and T2DM, with three to five repeated measurements for every participant. The present study aimed to investigate the relationship between pre- and post-diagnostic 25(OH)D concentrations and T2DM over a period of 30 years in individuals who developed T2DM compared to healthy controls.

## Materials and methods

### Study population

The Tromsø Study is an ongoing health survey based on the residents of the municipality of Tromsø in Northern Norway [19, 20]. Briefly, it was initiated in 1974, with surveys conducted approximately every 7 years; to-date, seven surveys have been completed (Tromsø1 through Tromsø7). At each survey, participants answered questionnaires, attended physical examinations, and had blood samples collected, which were frozen and stored as serum at − 70 °C.

**Fig. 1:**
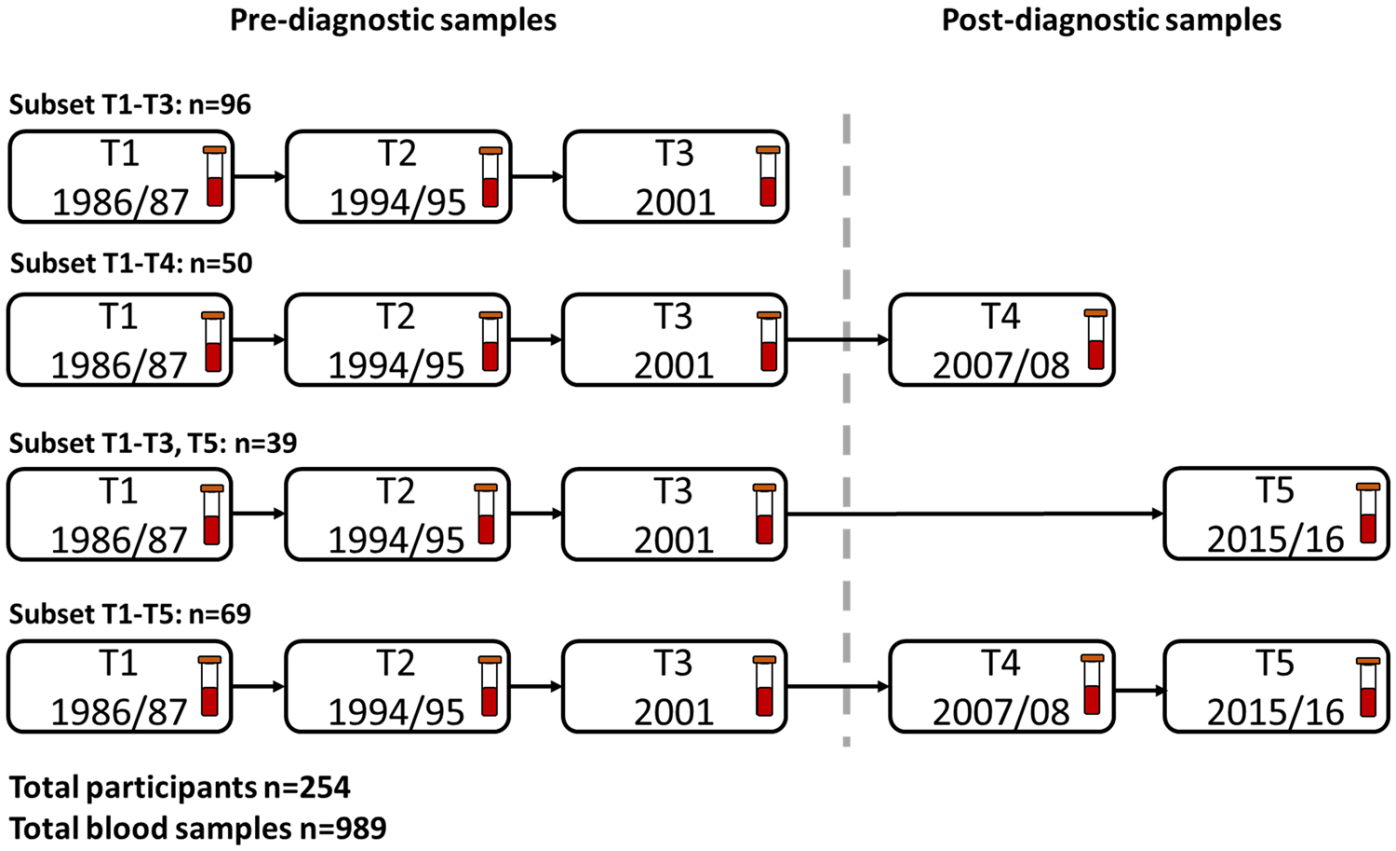
Overview of available serum samples and subsets based on participation in different surveys (time-points, T) of the Tromsø Study 1986–2016. The stippled line represents the separation between pre- and post-T2DM diagnostic time-points in cases

We used a longitudinal nested case–control design with repeated measurements from Tromsø3 (1986/87), Tromsø4 (1994/95), Tromsø5 (2001), Tromsø6 (2007/08), and Tromsø7 (2015/16), which we will refer to as time-points 1 through 5 (T1 through T5). The inclusion criteria for cases were T2DM diagnosis recorded in the local diabetes registry after the year 2000 (between T3 and T4), and available pre-diagnostic serum samples at T1, T2, and T3. Seventy-six women and 69 men met these criteria. Controls were randomly selected among those who had no T2DM diagnosis recorded in a local diabetes registry and then matched 1:1 by sex and participation in the same surveys as cases. In total, 290 participants were eligible for inclusion for T1–T3, of which 130 attended T4 and 122 attended T5 and had available serum samples. We excluded 29 cases with glycated haemoglobin (HbA_1c_) levels higher than 48 mmol/mol (6.5%) at T3 or earlier, and seven controls with HbA_1c_ levels higher than 48 mmol/mol at any time-point. The final sample included 254 participants at T1, T2, and T3, respectively, 119 at T4, and 108 at T5 (989 serum samples in total, Fig. 1). Informed consent was received at each survey from all the participants. The Regional Ethics Committee, REK, Nord approved the study protocol (REK reference: 2015/1780/REK Nord).

### Vitamin D analysis

Serum samples were randomised in batches within each time-point with equal amounts of cases, controls, men, and women, and were thawed and analysed for total 25(OH)D (hereafter referred to as 25(OH)D) over a period of 2 weeks at the Department of Laboratory Medicine, University Hospital of North Norway. Laboratory technicians were blinded to the sample number and time-point. The laboratory is a clinical laboratory accredited by the ISO 15189 standard and routinely runs vitamin D testing by liquid–liquid extraction (LLE) and liquid chromatography–tandem mass spectrometry (LC–MS/MS) detection, as described in detail elsewhere [21]. LLE was performed on a Tecan Fluent liquid handler (Männedorf, Switzerland), and LC–MS/MS detection was performed on a Waters Acquity™ *I*-class (Waters, Milford, MA) interfaced with Waters Xevo TQ-XS (Waters, Manchester, UK). MassCheck® quality control levels 1 and 2 for 25(OH)D (Chromsystems Instruments & Chemicals GmbH, München, Germany) were included with each batch, and the controls deviated less than 5% from the target values. The laboratory participates in an external proficiency programme (DEQAS, UK) and performs well within accepted target range values.

### Statistical analyses

25(OH)D concentrations and sample characteristics are reported as means with standard deviation (SD), and/or frequencies with percentages. Sample characteristics were compared between cases and controls at each time-point using independent two-sample *t* tests for continuous variables and Pearson’s *χ*^2^ test for categorical variables.

Potential confounding variables in the causal pathway between 25(OH)D and T2DM were identified by a directed acyclic graph (DAG, Fig. S1) [22], which indicated that the relationship could be confounded by age, body mass index (BMI), weight change between time-points (T1 set to zero), and physical activity (active: ≥ 3 h/week of light activity and/or ≥ 1 h hard exercise/week; inactive: < 3 h/week of activity that provoked perspiration or no activity). Month of blood sample collection (as a proxy for exposure to sunlight) and cod liver oil intake were not identified as confounders in the DAG. However, these variables varied by case–control status and time-point, and could have affected the time trends in 25(OH)D concentrations. Therefore, we added these two variables in the adjustment of time-trend analyses.

Linear mixed effects models were used to examine changes in 25(OH)D from T1 to T5, between and within cases and controls, after adjusting for DAG confounders, month of blood sample collection, and cod liver oil intake. 25(OH)D concentration (continuous) was used as the dependent variable; T2DM status, DAG confounders, month of blood sample collection, cod liver oil intake, and indicator variables of time with two-way interaction terms with T2DM status were used as independent variables. A random intercept at the participant level to control for repeated measurements over time, and an unstructured variance and covariance correlation structure for within-group errors was used. To fully explore the effect of month of blood collection, we repeated the same model, using month-specific 25(OH)D *z*-score as a dependent variable and removing month of blood collection as a confounder.

We used logistic regression to estimate odds-ratios (OR) for the association between 25(OH)D and T2DM at each time-point. We applied models with 25(OH)D as a continuous and dichotomised (< 50 nmol/l, i.e. vitamin D deficient and ≥ 50 nmol/l, i.e. vitamin D sufficient) independent variables, with T2DM status as the dependent variable, and adjusted for DAG confounders. To take advantage of the repeated measurements, we further calculated the area under the curve (AUC) for month-specific 25(OH)D *z*-score (to account for the variation in the month of blood sample collection between time-points) for pre-diagnostic samples. The AUC was then used as the independent variable in a logistic regression model along with DAG confounders measured at T1. Similarly, to explore associations between changes in 25(OH)D concentrations and T2DM in logistic regression models, we included the difference in month-specific 25(OH)D *z*-score (Δ25(OH)D) for each individual between T1 and T3, and between T3 and T5 as independent variables along with DAG confounders from T1 and T3, respectively.

Statistical analyses were performed in STATA (v 17.0, StataCorp LLC, 4905 Lakeway Drive, College Station, Texas USA). Significance was set at 5%, and *p* values were two-sided. All analyses were stratified by sex.

## Results

### Study sample characteristics

Cases and controls were similar in age and experienced the same weight change throughout the study period (Table 1). Cases were significantly heavier and had higher BMI at all time-points. Cases and controls had a similar physical activity level and cod liver oil intake, except for women at T2 and T5, and men at T5, where controls were more active, and at T3, where women controls had higher cod liver oil intake. Month of blood sample collection was similar for cases and controls at each time-point (*p* values 0.11–0.97), but varied between time-points (*p* values < 0.01). At T1, more blood samples were collected from December to February and from September to November, whereas at other time-points, blood sample collection was distributed more evenly across the year (Table S1). At T2, only 10 blood samples were collected from June to August, and at T3, only four blood samples were collected from December to February.

**Table 1:** Selected characteristics of cases and controls by time-point. The Tromsø Study 1986–2016

	T1 (1986/87)		T2 (1994/95)		T3 (2001)		T4 (2007/08)		T5 (2015/16	
	Mean (SD)	ΔMean case–control (95% CI)	Mean (SD)	ΔMean case–control (95% CI)	Mean (SD)	ΔMean case–control (95% CI)	Mean (SD)	ΔMean case–control (95% CI)	Mean (SD)	ΔMean case–control (95% CI)
*Age (years)*										
Women										
Case	46.3 (6.36)	2.50 (−0.19, 5.19)	54.3 (6.36)	2.50 (−0.19, 5.19)	61.3 (6.36)	2.50 (−0.19, 5.19)	66.8 (6.55)	1.14 (−1.58, 3.87)	74.1 (6.14)	3.55 (−0.38, 7.49)
Control	43.8 (8.88)		51.8 (8.88)		58.8 (8.88)		65.7 (7.71)		70.5 (9.47)	
Men										
Case	48.8 (8.66)	2.09 (−1.49, 5.64)	56.8 (8.66)	2.09 (−1.49, 5.64)	63.8 (8.66)	2.09 (−1.49, 5.64)	69.1 (7.22)	1.92 (−2.01, 5.85)	73.3 (8.42)	3.12 (−2.66, 8.90)
Control	46.7 (10.7)		54.7 (10.7)		61.7 (10.7)		67.2 (10.1)		70.2 (11.0)	
*BMI (kg/m*^*2*^*)*										
Women										
Case	27.1 (4.27)	3.35** (1.97, 4.72)	29.2 (4.91)	4.21** (2.58, 5.83)	31.2 (5.69)	4.88** (3.12, 6.63)	31.3 (6.09)	4.66** (2.54, 6.78)	31.4 (6.85)	4.56* (1.59, 7.53)
Control	23.7 (3.75)		25.0 (4.61)		26.4 (4.60)		26.6 (5.04)		26.8 (5.76)	
Men										
Case	27.6 (3.49)	2.92** (1.79, 4.05)	28.7 (3.44)	3.01** (1.84, 4.18)	29.8 (3.52)	3.28** (2.02, 4.55)	29.5 (3.38)	2.51** (1.01, 4.00)	29.4 (3.74)	2.20* (0.11, 4.29)
Control	24.7 (2.72)		25.7 (3.02)		26.6 (3.44)		26.9 (3.25)		27.2 (3.49)	
*Weight (kg)*										
Women										
Case	70.8 (11.6)	7.95** (4.28, 11.6)	76.2 (13.1)	10.2** (5.96, 14.4)	80.6 (14.5)	11.5** (6.97, 16.0)	80.6 (15.1)	11.9** (6.55, 17.2)	81.3 (17.3)	11.8** (4.15, 19.5)
Control	62.9 (9.89)		66.0 (11.7)		69.1 (12.0)		68.8 (13.0)		69.4 (15.2)	
Men										
Case	85.5 (12.9)	7.11** (3.08, 11.1)	88.8 (13.3)	7.66** (3.41, 11.9)	91.6 (13.8)	8.01** (3.39, 12.6)	90.7 (11.8)	5.94* (0.84, 11.0)	89.7 (14.3)	3.71 (−3.74, 11.2)
Control	78.3 (9.16)		81.2 (10.1)		83.6 (11.6)		84.8 (10.8)		86.0 (11.7)	
*Weight change (kg)*										
Women										
Case	NA	NA	5.36 (4.34)	2.25* (0.68, 3.82)	4.44 (5.47)	1.30 (−0.70, 3.29)	−0.57 (5.77)	−1.12 (−3.24, 0.99)	−1.55 (5.93)	−1.38 (−4.12, 1.37)
Control	NA	NA	3.11 (4.73)		3.14 (6.04)		0.55 (5.39)		-0.17 (5.15)	
Men										
Case	NA	NA	3.38 (4.07)	0.55 (−1.04, 2.14)	2.79 (5.20)	0.34 (−1.66, 2.35)	−2.46 (6.10)	−2.44 (−5.02, 0.15)	−1.05 (8.26)	−1.98 (−6.36, 2.40)
Control	NA	NA	2.83 (4.64)		2.44 (5.78)		−0.03 (5.34)		0.93 (6.12)	
*25(OH)D concentration (nmol/l)*										
Women										
Case	44.8 (12.6)	−1.18 (−5.99, 3.62)	53.6 (14.9)	−2.85 (−7.88, 2.18)	46.6 (13.6)	−13.5** (−19.3, −7.72)	50.9 (11.5)	−11.4* (−21.6, -1.34)	69.7 (24.6)	1.06 (−11.5, 13.6)
Control	46.0 (15.1)		56.4 (14.5)		60.1 (19.1)		62.3 (27.9)		68.6 (24.6)	
Men										
Case	53.4 (17.1)	6.46* (0.04, 12.6)	58.5 (16.4)	2.39 (−3.28, 8.07)	53.3 (13.6)	−5.22 (−10.8, 0.32)	59.7 (26.5)	7.42 (−5.96, 20.8)	67.5 (22.6)	8.73 (−3.70, 21.2)
Control	47.0 (16.5)		56.1 (14.8)		58.5 (16.5)		52.3 (15.9)		58.8 (16.2)	

	*n* (%)	*p* value	* n* (%)	*p *value	* n* (%)	*p *value	*n* (%)	*p* value	* n* (%)	*p* value
*Physical activity: women*										
Active										
Case	48 (80.0)	0.71	26 (43.3)	0.004	44 (74.6)	0.64	33 (73.3)	0.07	19 (73.1)	0.05
Control	58 (77.3)		51 (68.0)		57 (78.1)		44 (88.0)		33 (91.7)	
Inactive										
Case	12 (20.0)		34 (56.7)		15 (25.4)		12 (26.7)		7 (26.9)	
Control	17 (22.7)		24 (32.0)		16 (21.9)		6 (12.0)		3 (8.3)	
*Physical activity: men*										
Active										
Case	46 (82.1)	0.96	39 (69.6)	0.55	42 (77.8)	0.62	28 (71.8)	0.51	9 (45.0)	<0.001
Control	52 (82.5)		47 (74.6)		45 (73.8)		29 (78.4)		24 (92.3)	
Inactive										
Case	10 (17.9)		17 (30.4)		12 (22.2)		11 (28.2)		11 (55.0)	
Control	11 (17.5)		16 (25.4)		16 (26.2)		8 (21.6)		2 (7.7)	
*Cod liver oil intake: women*										
No										
Case	40 (81.6)	0.16	29 (58.0)	0.21	25 (44.6)	0.01	23 (54.8)	0.96	19 (67.9)	0.08
Control	47 (70.2)		29 (46.0)		17 (23.3)		26 (55.3)		16 (45.7)	
Yes										
Case	9 (18.4)		21 (42.0)		31 (55.4)		19 (45.2)		9 (32.1)	
Control	20 (29.9)		34 (54.0)		56 (76.7)		21 (44.7)		19 (54.3)	
*Cod liver oil intake: men*										
No										
Case	33 (71.7)	0.98	26 (50.0)	0.86	26 (54.2)	0.1	17 (50.0)	0.37	14 (73.7)	0.25
Control	41 (71.9)		30 (51.7)		23 (38.3)		23 (60.5)		16 (57.1)	
Yes										
Case	13 (28.3)		26 (50.0)		22 (45.8)		17 (50.0)		5 (26.3)	
Control	16 (28.1)		28 (48.3)		37 (61.7)		15 (39.5)		12 (42.9)	
*Vitamin D status (nmol/l): women*										
>50										
Case	21 (35.0)	0.23	32 (53.3)	0.21	21 (35.0)	<0.001	17 (48.6)	0.02	21 (72.4)	0.09
Control	34 (45.3)		48 (64.0)		51 (68.9)		29 (74.4)		25 (75.8)	
<50										
Case	39 (65.0)		28 (46.7)		39 (65.0)		18 (51.4)		8 (27.6)	
Control	41 (54.7)		27 (36.0)		23 (31.1)		10 (25.6)		8 (24.2)	
*Vitamin D status (nmol/l): men*										
>50										
Case	33 (58.9)	0.02	39 (69.6)	0.98	30 (53.6)	0.15	15 (71.4)	0.15	15 (83.3)	0.27
Control	24 (38.1)		44 (69.8)		42 (66.7)		11 (50.0)		15 (68.2)	
<50										
Case	23 (41.1)		17 (30.4)		26 (46.4)		6 (28.6)		3 (16.7)	
Control	39 (61.9)		19 (30.2)		21 (33.3)		11 (50.0)		7 (31.8)	

Weight change was calculated between time-points (T1 set to zero). Pre-diagnostic period: T1 to T3. Post-diagnostic period: T4 to T5

*25(OH)D* 25-hydroxyvitamin D, *CI* confidence interval, *SD* standard deviation, *T* time-point

*p value < 0.05, **p value < 0.01

### Vitamin D concentrations

In women, cases had lower 25(OH)D concentrations than controls at every time-point (significantly at T3 and T4) except T5, when concentrations were similar (Table 1). In men, cases had higher 25(OH)D concentrations than controls at all time-points (significantly at T1) except T3, when concentrations were lower in cases. Among women, there was a significantly higher percentage of cases than controls with insufficient vitamin D status at T3 and T4. For men, there was a significantly higher percentage of controls with insufficient vitamin D status at T1 (Table 1).

### Longitudinal changes in vitamin D

**Fig. 2:**
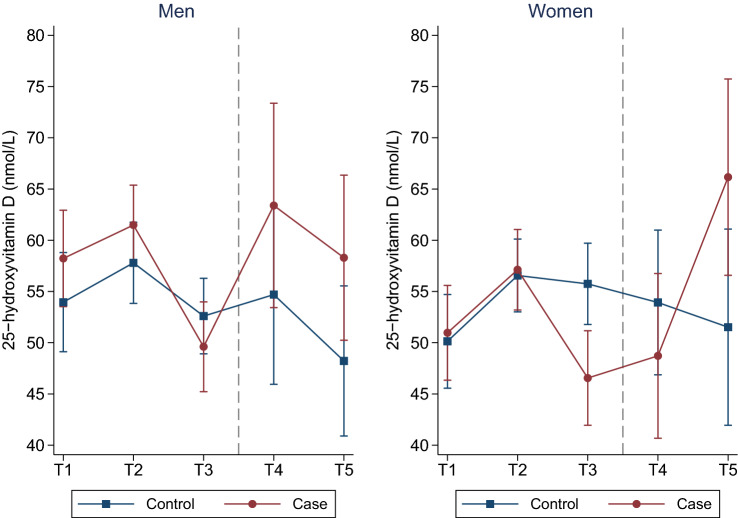
Estimated mean 25-hydroxyvitamin D concentrations (*y*-axis) across five time-points for cases and controls. Models were adjusted for age, BMI, weight change, physical activity, month of blood sample collection, and cod liver intake. The Tromsø Study 1986–2016. *T* time-point. Dots/squares represent mean concentrations and whiskers the 95% confidence interval around the mean

After adjusting for DAG confounders (age, BMI, weight change, physical activity), month of blood sample collection and cod liver oil intake, all participants’ 25(OH)D concentrations increased from T1 to T2, followed by a decrease from T2 to T3 (Fig. 2 and Table S2). Cases experienced a significantly larger decrease in 25(OH)D concentrations from T2 to T3 compared to controls. Further, post-diagnostic (T3 to T5) 25(OH)D concentrations increased in cases compared to controls; the latter experienced an overall decrease. Repeating the analyses using month-specific 25(OH)D *z*-scores yielded similar results (results not presented).

### Associations between vitamin D and T2DM

At T1, a 5-nmol/l increase in 25(OH)D concentration was associated with 15% higher odds (OR 1.15, 95% CI 1.00, 1.31) for T2DM in men. Likewise, sufficient vitamin D status was positively associated with T2DM compared to insufficient vitamin D status (OR 2.98, 95% CI 1.24, 7.17). In women, a 5-nmol/l increase in 25(OH)D concentration was associated with 21% lower odds of T2DM (OR 0.79, 95% CI 0.68, 0.91) at T3. At the same time-point, sufficient vitamin D status was inversely associated with T2DM compared to insufficient vitamin D status (OR 0.29, 95% CI 0.13, 0.69) (Table 2). At all other time-points, neither 25(OH)D concentrations nor vitamin D status was significantly associated with T2DM. Results were similar when repeating the analyses with month-specific 25(OH)D *z*-scores (results not presented).

**Table 2:** ORs with 95% CIs for the associations between 25(OH)D concentrations and T2DM in women and men. The Tromsø Study 1986–2016

		T1 (1986/87)		T2 (1994/95)		T3 (2001)		T4 (2007/08)		T5 (2015/16)	
		Crude	Adjusted^a^	Crude	Adjusted^a^	Crude	Adjusted^a^	Crude	Adjusted^a^	Crude	Adjusted^a^
Biomarker	Sex	OR (95% CI)	OR (95% CI)	OR (95% CI)	OR (95% CI)	OR (95% CI)	OR (95% CI)	OR (95% CI)	OR (95% CI)	OR (95% CI)	OR (95% CI)
25(OH)D (per 5-nmol/l increase)	Women	0.97 (0.86, 1.10)	1.00 (0.87, 1.15)	0.93 (0.83, 1.05)	0.97 (0.84, 1.12)	0.77* (0.68, 0.87)	0.79* (0.68, 0.91)	0.79* (0.65, 0.96)	0.79 (0.60, 1.02)	1.01 (0.91, 1.12)	1.06 (0.93, 1.22)
	Men	1.12* (1.00, 1.26)	1.15* (1.00, 1.31)	1.05 (0.94, 1.18)	1.09 (0.95, 1.26)	0.89 (0.79, 1.01)	0.90 (0.76, 1.07)	1.09 (0.93, 1.28)	1.14 (0.96, 1.35)	1.13 (0.95, 1.35)	1.21 (0.89, 1.65)
25(OH)D (> 50 nmol/l)^b^	Women	0.65 (0.32, 1.31)	0.65 (0.30, 1.40)	0.64 (0.32, 1.28)	0.75 (0.35, 1.70)	0.24* (0.12, 0.50)	0.29* (0.13, 0.69)	0.33* (0.12, 0.87)	0.25 (0.06, 1.03)	1.84 (0.27, 2.62)	1.03 (0.21, 5.05)
	Men	2.33* (1.12, 4.87)	2.98* (1.24, 7.17)	0.99 (0.45, 2.17)	1.23 (0.46, 3.25)	0.58 (0.27, 1.21)	0.86 (0.32, 2.30)	2.50 (0.71, 8.84)	3.83 (0.75, 19.4)	2.33 (0.51, 10.8)	23.4 (0.74, 743)

25*(OH)D* 25-hydroxyvitamin D, *CI* confidence interval, *OR* odds-ratio, *T* time-point

*p value < 0.05

^a^Adjusted for age, body mass index, weight change, and physical activity

^b^< 50 nmol/l is set as the reference

Each 1-unit increase in the pre-diagnostic difference (T3 to T1) in month-specific Δ25(OH)D *z*-score was significantly and inversely associated with T2DM in both sexes, whereas each 1-unit increase in post-diagnostic difference (T5 to T4) was significantly associated with higher odds of T2DM in women (Table 3). There were no significant associations between pre-diagnostic AUC for 25(OH)D *z*-score and T2DM.

**Table 3:** ORs with 95% CIs for the associations between month-specific 25(OH)D *z*-score (as summary variable) and T2DM in women and men. The Tromsø Study 1986–2016

	T1–T3				T3–T5	
	Δ25(OH)D^a^		AUC 25(OH)D^b^		Δ25(OH)D^c^	
	Crude OR (95% CI)	Adjusted^d^ OR (95% CI)	Crude OR (95% CI)	Adjusted^d^ OR (95% CI)	Crude OR (95% CI)	Adjusted^e^ OR (95% CI)
Women	0.45* (0.29, 0.70)	0.51* (0.32, 0.80)	0.80 (0.63, 1.02)	0.85 (0.64, 1.12)	2.21* (1.37, 3.56)	2.48* (1.39, 4.43)
Men	0.48* (0.31, 0.74)	0.52* (0.33, 0.84)	1.07 (0.85, 1.34)	1.23 (0.88, 1.57)	2.09* (1.05, 4.18)	1.93 (0.90, 4.12)

*25(OH)D* 25-hydroxyvitamin D, *CI* confidence interval, *OR* odds-ratio, *T* time-point

*p value < 0.05

^a^Change in month-specific 25(OH)D *z*-score from T1 to T3

^b^AUC for month-specific 25(OH)D *z*-scores for pre-diagnostic samples

^c^Change in month-specific 25(OH)D *z*-score from T3 to T5

^d^Adjusted for age, body mass index, weight change, and physical activity at T1

^e^Adjusted for age, body mass index, weight change, and physical activity at T3

## Discussion

This is the first observational study with repeated pre-and post-diagnostic 25(OH)D concentrations in T2DM cases and controls over a 30-year time period. Our results suggest that there is an association between changes in 25(OH)D concentrations and T2DM. This is supported by our findings that: (1) cases and controls had similar 25(OH)D concentrations (higher for cases at T1 for men) 7–15 years prior to diagnosis; (2) cases experienced significantly larger pre-diagnostic declines closer to the time of diagnosis, and (3) cases had substantial post-diagnostic increases in 25(OH)D concentrations compared to controls. As a result, decreases in 25(OH)D concentrations in the pre-diagnostic period were inversely associated with T2DM, whereas increases in the post-diagnostic period were positively associated with T2DM. It is likely that pre-diagnostic 25(OH)D concentrations are affected by factors related to disease progression and dietary habits, whereas post-diagnostic concentrations could be impacted by an overall improvement in health following T2DM diagnosis (e.g. dietary counselling and medication). This is supported by our previous findings in this population, where cases significantly improved their lipid profiles after diagnosis [23].

Mendelian randomisation studies and intervention studies have addressed the causal relationship between vitamin D and T2DM but with inconclusive and/or non-significant results [12, 16, 24, 25]. Rejnmark et al. [26] summarised findings from observational studies and concluded that the progression of a large number of diseases, including T2DM, is associated with low vitamin D concentrations; however, intervention studies of vitamin D supplementation on these diseases did not provide causal evidence. A common denominator for diseases associated with low vitamin D concentrations is underlying inflammation [27]. Palaniswami et al. [28] observed a significant association between low vitamin D status and inflammation; however, they reported that neither their Mendelian randomisation analysis nor their review of randomised controlled trials (RCTs) supported a causal relationship. Likewise, a review article by Cannell et al. [29] summarised evidence from RCTs and concluded that several studies reported reduced inflammation with higher vitamin D status. Still, it is not clear if vitamin D can lower inflammation or if inflammation can lower vitamin D. Clearly, the relationship between vitamin D, inflammation, and T2DM is complex, and the order of events prior to disease diagnosis is unclear. Nevertheless, studies comparing vitamin D supplementation vs placebo have consistently reported non-significant risk reductions for T2DM in the vitamin D supplement group, which prompts the use of vitamin D supplements in individuals at high risk for T2DM [16].The potential benefits of vitamin D supplementation are supported by Lemieux et al. [30] in an intervention study that showed significant improvements in insulin sensitivity and beta-cell function for individuals at high risk of T2DM or had newly diagnosed T2DM.

Our study showed that the associations between 25(OH)D concentrations and T2DM were different in men and women. Around 15 years prior to diagnosis, a positive association between 25(OH)D concentrations and T2DM was observed in men, whereas in women, 25(OH)D concentrations were inversely associated with T2DM at all pre-diagnostic time-points, although they were only significant at the time-point closest to diagnosis (T3) in cases. Wierzbicka et al. [6] discusses several sex-specific factors that may influence vitamin D status differently in men and women, of which per cent body fat and sex hormones play a role in circulating vitamin D levels. They noted that higher testosterone and oestrogens levels in men and women, respectively, were significantly associated with higher vitamin D levels, and that women, who generally have a higher percentage of body fat than men, often have lower circulating vitamin D levels than men. In line with our findings, Schöttker et al. [31] found a significant association between low vitamin D status in women and increased risk of T2DM. Further, most studies observed either an increased risk of T2DM with lower 25(OH)D concentrations [13, 15, 25, 32–36], or non-significant associations [37–40]. To our knowledge, no previous studies have reported positive associations between 25(OH)D concentrations and T2DM.

Inconsistencies across studies could be explained by the complexity of the relationship between vitamin D and T2DM, the slow progression and heterogeneity of the disease, and different follow-up times. This clearly emphasises the importance of repeated measurements that capture variations in 25(OH)D concentrations over time. There are three other studies that included repeated measurements, and, like us, they observed that decreased vitamin D concentrations in the pre-diagnostic period was associated with increased risk of T2DM [14, 15, 41]. The variability in vitamin D concentrations from one time-point to another has been investigated previously in the Tromsø Study. Kubiak et al. [42] reported 25(OH)D concentrations from three time-points over a 21-year period and observed a decrease in the correlation between 25(OH)D concentrations in the same individuals over time. They also identified that change in cod liver oil/vitamin D supplement intake and BMI were important factors for changes in vitamin D status between time-points. As 25(OH)D concentrations are affected by lifestyle habits, which may change greatly throughout an individual’s lifetime, a design with repeated measurements from prospective T2DM cases and controls will yield more accurate conclusions about vitamin D and T2DM than studies relying on blood samples collected at one point in time. Accordingly, a major strength of this study is its design, with up to five repeated measurements in cases and controls over a period of 30 years. T2DM diagnosis was ascertained in local registries, and laboratory data and medical records confirmed the absence of T2DM among controls. All 25(OH)D measurements were analysed from thawed serum by LC–MS/MS using accredited standards. However, the observational nature of this study does not allow for causal inference and the precision of our estimates might have been affected by stratifying by sex. We also had fewer blood samples at post-diagnostic time-points, which further affects the precision of estimates at T4 and T5. T2DM diagnosis did not vary over time, but was set at T3 for all cases, which meant we were unable to fully integrate the longitudinal relationship in the logistic regression models [43].

We believe that the generalisability of our results to other populations improves by adjusting for proper confounders that are specific for the Northern Norwegian population such as seasonal variation in sun exposure and dietary intake of cod liver oil and vitamin D supplements, as increased intake of vitamin D from these sources during the winter months reduces the effect of season. Hence, vitamin D concentrations in Norway do not fluctuate by season as much as they do in countries located further south [42, 44, 45].

## Conclusion

Our results indicate that pre-diagnostic decreases in vitamin D concentrations are associated with T2DM progression and diagnosis, whereas post-diagnostic increases in concentrations are influenced by intervention and treatment efforts.

## Supplementary Information

Below is the link to the electronic supplementary material.

Supplementary file1 (DOCX 96 KB)

## Data Availability

The dataset in this study, which is not publicly available, was acquired from the Tromsø Study. It may be accessed through an application to the Tromsø Study (https://uit.no/research/tromsostudy).
