# Supplementary material for: Longitudinal changes in vitamin D concentrations and the association with type 2 diabetes mellitus: the Tromsø Study
Source: Acta Diabetol. 2022 Dec 2;60(2):293–304. doi: 10.1007/s00592-022-02001-y (PMC9852201; doi:10.1007/s00592-022-02001-y)
Supplement: Supplementary file 1 — Supplementary file1 (DOCX 96 KB) [file 592_2022_2001_MOESM1_ESM.docx]

**Supplemental material**

**Longitudinal changes in vitamin D concentrations and the association with type 2 diabetes mellitus – The Tromsø Study**

Giovanni Allaoui^ab^, Charlotta Rylander^c^, Ole-Martin Fuskevåg^ad^, Maria Averina^ad^, Tom Wilsgaard^c^, Magritt Brustad^ce^, Rolf Jorde^d^, Vivian Berg^ab*^

^a^Department of Laboratory Medicine, Diagnostic clinic, University Hospital of North – Norway, NO-9038 Tromsø, Norway

^b^Department of Medical Biology, Faculty of Health Sciences, UiT-The Arctic University of Norway, NO-9037 Tromsø, Norway

^c^Department of Community Medicine, Faculty of Health Sciences, UIT-The Arctic University of Norway, NO-9037 Tromsø, Norway

^d^Department of Clinical Medicine, Tromsø Endocrine Research Group, Uit-The Arctic University of Norway, NO-9037 Tromsø, Norway

^e^The Public Dental Health Service Competence Centre of Northern Norway (TkNN), NO-9019 Tromsø, Norway

***Correspondence:**

Vivian Berg, Department of Medical Biology, Faculty of Health Sciences, UiT-The Arctic University of Norway, NO-9037 Tromsø, Norway

Phone: +47 77 64 46 77

E-mail: vivian.berg@uit.no

Table S1. Month of blood sample collection in women and men by time-point. The Tromsø Study 1986-2016.

|  |  |  | **Pre-diagnostic time-points** | | | **Post-diagnostic time-points** | | |
| --- | --- | --- | --- | --- | --- | --- | --- | --- |
|  |  |  | **T1 (1986/87)** | **T2 (1994/95)** | **T3 (2001)** | **T4 (2007/08)** | **T5 (2015/16)** |  |
|  |  |  | n (%) | n (%) | n (%) | n (%) | n (%) |  |
| Month of blood sample collection: Women | January  February  March  April  May  June  July  August  September  October  November  December | Case  Control  Case  Control  Case  Control  Case  Control  Case  Control  Case  Control  Case  Control  Case  Control  Case  Control  Case  Control  Case  Control  Case  Control | 11 (18.3)  17 (22.7)  8 (13.3)  12 (16.0)  10 (16.7)  7 (9.3)  0 (0.0)  3 (4.0)  NA  NA  NA  NA  NA  NA  1 (1.7)  0 (0.0)  5 (8.3)  6 (8.0)  11 (18.3)  12 (16.0)  10 (16.7)  11 (14.7)  4 (6.7)  7 (9.3) | 11 (18.3)  14 (18.7)  6 (10.0)  2 (2.7)  6 (10.0)  10 (13.3)  4 (6.7)  7 (9.3)  7 (11.7)  10 (13.3)  1 (1.7)  5 (6.7)  NA  NA  NA  NA  4 (6.7)  6 (8.0)  6 (10.0)  2 (2.7)  7 (11.7)  15 (20.0)  8 (13.3)  4 (5.3) | NA  NA  NA  NA  5 (8.3)  5 (6.7)  4 (6.7)  13 (17.3)  8 (13.3)  10 (13.3)  8 (13.3)  9 (12.0)  NA  NA  2 (3.3)  8 (10.7)  9 (15.0)  5 (6.7)  13 (21.7)  16 (21.3)  11 (18.3)  6 (8.0)  0 (0.0)  3 (4.0) | 7 (13.5)  6 (10.5)  3 (5.8)  4 (7.0)  4 (7.7)  8 (14.0)  4 (7.7)  6 (10.5)  3 (5.8)  3 (5.3)  7 (13.5)  2 (3.5)  NA  NA  5 (9.6)  4 (7.0)  8 (15.4)  9 (15.8)  7 (13.5)  6 (10.5)  0 (0.0)  8 (14.0)  4 (7.7)  1 (1.8) | 1 (3.3)  5 (11.9)  1 (3.3)  4 (9.5)  0 (0.0)  5 (11.9)  3 (10.0)  2 (4.8)  4 (13.3)  6 (14.3)  5 (16.7)  5 (11.9)  NA  NA  2 (6.7)  5 (11.9)  5 (16.7)  5 (11.9)  2 (6.7)  2 (4.8)  5 (16.7)  1 (2.4)  2 (6.7)  2 (4.8) |  |
| Month of blood sample collection: Men | January  February  March  April  May  June  July  August  September  October  November  December | Case  Control  Case  Control  Case  Control  Case  Control  Case  Control  Case  Control  Case  Control  Case  Control  Case  Control  Case  Control  Case  Control  Case  Control | 13 (23.2)  11 (17.5)  11 (19.6)  8 (12.7)  7 (12.5)  14 (22.2)  2 (3.6)  3 (4.8)  NA  NA  NA  NA  NA  NA  0 (0.0)  1 (1.6)  6 (10.7)  4 (6.4)  8 (14.3)  5 (7.9)  6 (10.7)  8 (12.7)  3 (5.4)  9 (14.3) | 9 (16.1)  19 (30.2)  7 (12.5)  8 (12.7)  5 (8.99  2 (3.2)  4 (7.14)  8 (12.7)  9 (16.1)  6 (9.5)  0 (0.0)  4 (6.4)  NA  NA  NA  NA  8 (14.3)  4 (6.4)  4 (7.14)  4 (6.4)  6 (10.7)  2 (3.2)  4 (7.14)  6 (9.5) | NA  NA  NA  NA  4 (7.1)  3 (4.8)  6 (10.7)  3 (4.8)  14 (25.0)  9 (14.3)  5 (8.9)  10 (15.9)  NA  NA  3 (5.4)  3 (4.8)  7 (12.5)  13 (20.6)  11 (19.6)  8 (12.7)  6 (10.7)  13 (20.6)  0 (0.0)  1 (1.6) | 2 (5.0)  4 (10.3)  1 (2.5)  2 (5.13)  5 (12.5)  3 (7.7)  4 (10.0)  3 (7.7)  2 (5.0)  3 (7.7)  4 (10.0)  4 (10.3)  NA  NA  9 (22.5)  6 (15.4)  2 (5.0)  3 (7.7)  2 (5.0)  3 (7.7)  4 (10.0)  5 (12.8)  5 (12.5)  3 (7.7) | 0 (0.0)  2 (7.1)  1 (4.8)  2 (7.1)  1 (4.8)  3 (10.7)  2 (9.5)  1 (3.6)  3 (14.3)  2 (7.1)  1 (4.8)  2 (7.1)  2 (9.5)  1 (3.6)  1 (4.8)  3 (10.7)  3 (14.3)  3 (10.7)  1 (4.8)  5 (17.9)  5 (23.8)  4 (14.3)  1 (4.8)  0 (0.0) |  |

T, time-point.

**Table S2.** Linear mixed effects models adjusted for age, BMI, weight change, physical activity, month of blood sample collection, and cod liver oil intake. T3 is set as the reference time-point.

| **Biomarker** |  | **Men** | | | **Women** | | |
| --- | --- | --- | --- | --- | --- | --- | --- |
|  |  | **β-coefficient**  **(SE)** | **p-value** | **95% confidence interval** | **β-coefficient**  **(SE)** | **p-value** | **95% confidence interval** |
| **Total 25(OH)D** (nmol/l) | Case  T1  T2  T3  T4  T5  Case#T1  Case#T2  Case#T3  Case#T4  Case#T5  Constant | -3.00 (2.79)  1.36 (2.83)  5.20 (2.27)  Reference  2.10 (4.32)  -4.38 (3.66)  7.27 (3.07)  6.68 (2.84)  Reference  11.7 (6.25)  13.1 (4.95)  28.7 (11.5) | 0.28  0.63  0.02  --  0.63  0.23  0.02  0.02  --  0.06  0.01  0.01 | -8.47, 2.48  -4.19, 6.91  0.75, 9.65  --  -6.37, 10.6  -11.5, 2.79  1.25, 13.3  1.12, 12.3  --  -0.56, 23.9  3.37, 22.8  6.10, 51.3 | -9.19 (3.03)  -5.61 (2.73)  0.82 (2.21)  Reference  -1.82 (3.13)  -4.23 (4.64)  10.0 (2.72)  9.75 (2.94)  Reference  3.98 (4.37)  23.8 (5.83)  47.5 (9.05) | <0.01  0.04  0.71  --  0.56  0.36  <0.01  <0.01  --  0.36  <0.01  <0.01 | -15.1, -3.25  -11.0, -0.25  -3.50, 5.14  --  -7.96, 4.32  -13.3, 4.86  4.68, 15.4  3.99, 15.5  --  -4.58, 12.5  12.4, 35.3  29.7, 65.2 |

25(OH)D, 25-hydroxyvitamin D; T, time-point.


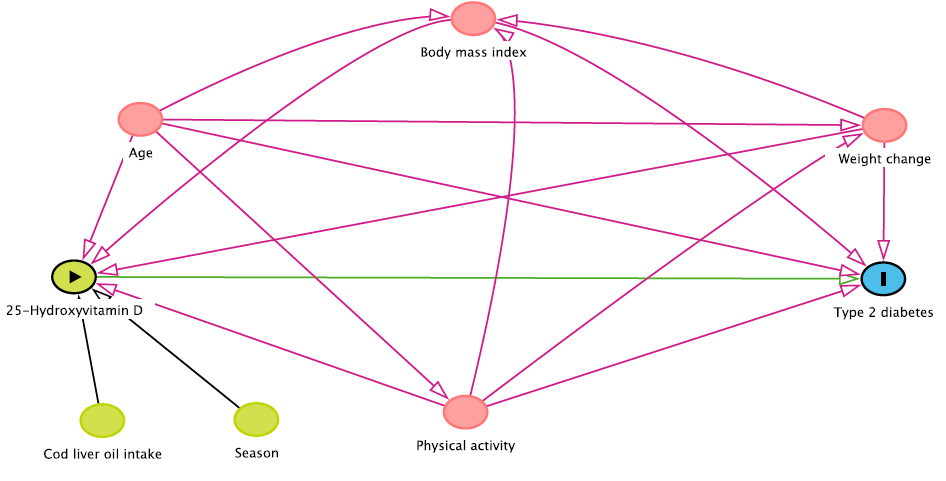


**Figure S1.** Directed acyclic graph illustrating assumptions about the associations and causal relationships between 25-hydroxyvitamin D and type 2 diabetes. Red circles illustrate confounders, green illustrate exposure, and blue is the outcome.
